# Novel coronavirus disease 2019: knowledge, practice and preparedness: a survey of healthcare workers in the Offinso-North District, Ghana

**DOI:** 10.11604/pamj.supp.2020.35.2.23644

**Published:** 2020-06-16

**Authors:** Charles Nkansah, Dorcas Serwaa, Louisa Akua Adarkwah, Felix Osei-Boakye, Kofi Mensah, Patrick Tetteh, Salima Awudu, Atorobah Apodola

**Affiliations:** 1 Department of Medical Diagnostic, Faculty of Allied Health Sciences, Kwame Nkrumah University of Science and Technology, Kumasi, Ghana; 2 Nkenkaasu District Hospital, Nkenkaasu, Ghana; 3 Reproductive Biology Unit, Department of Obstetrics and Gynaecology, College of Medicine, Pan African University of Life and Earth Sciences Institute (PAULESI), University of Ibadan, Ibadan, Nigeria; 4 Clinical Laboratory Department, Mankranso District Hospital, Mankranso, Ghana; 5 Clinical Laboratory Department, Komfo Anokye Teaching Hospital, Kumasi, Ghana; 6 Department of Physician Assistantship, Faculty of Health and Medical Science, Presbyterian University College of Ghana, Ghana; 7 Department of Mathematics, Faculty of Physical and Computational Sciences, Kwame Nkrumah University of Science and Technology, Kumasi, Ghana; 8 Department of Haematology, College of Medicine and Dentistry, University of Ghana, Ghana; 9 Department of Community Medicine, College of Health, Yamfo, Ghana

**Keywords:** Coronavirus disease 2019, knowledge, practice, preparedness, Ghana

## Abstract

**Introduction:**

Ever since World Health Organization declared COVID-19 as a public health emergency of international concern, Ghana is one of the most affected countries in Africa. However, the knowledge, practice, and preparedness of healthcare providers on the novel COVID-19 in the country has not been studied. This study assessed health workers’ knowledge, practice, and preparedness on the current pandemic in three Ghanaian hospitals.

**Methods:**

This multi-centre cross-sectional survey recruited 261 healthcare workers in Offinso North district between April and May, 2020, through self-administered questionnaire. SPSS version 22.0 was used for the analysis and statistical significance set at p < 0.05.

**Results:**

We identified that 65.1% of the healthcare providers had adequate knowledge, 27.6% of them had received appropriate in-service training, 57.5% had adequately prepared themselves and willing to care for affected patients and 63.0% were duly aware of their facility’s preparedness for the situation. Again, only 57.5% of the health workers adhered strictly to the practice of precautionary measures, and 62.5% had varying forms of misconceptions about the aetiology of the novel COVID-19. Majority (77.1%) of the respondents received regular information on the COVID-19 from the Ministry of Health, Ghana.

**Conclusion:**

The overall knowledge, practice and willingness of healthcare workers to handle COVID-19 were encouraging; however, this study still elucidates the knowledge gap of the professionals on the pandemic. We advocate for progressive training of staff on COVID-19, to advance the appreciation of the risks and precautionary measures and clear the misconceptions. This, we believe, would boost the confidence and increase their willingness to deliver efficiently.

## Introduction

The novel Coronavirus disease (COVID-19) caused by the seventh member of the family of coronaviruses that infect humans, SARS-CoV-2, is an evolving respiratory disease which was first detected in December, 2019 in Wuhan, China, and has eventually spread to larger parts of the world [1]. World Health Organization (WHO) on 30th January, 2020 declared the outbreak as a public health emergency of international concern and described it as pandemic on March 11, same year [2]. As of 16th May, 2020, the highly contagious disease had infected 4,647,960 people globally with loss of 308,985 lives; Africa had recorded 80,171 COVID-19 cases and 2,653 deaths, with Ghana recording over 5,638 cases and 28 deaths. These presentations are similar to the severe acute respiratory syndrome (SARS)-CoV and the Middle East respiratory syndrome (MERS)-CoV which were reported in 2002 and 2012 respectively [4]. COVID-19 may result in mild to severe respiratory distress, depending on the individuals´ age and immune system as well as the presence of any underlying conditions. This was evident in the [5] study which revealed that instead of the median incubation period of 14 days, patients older than 70 years had a shorter incubation period of the condition compared to younger patients. The WHO earlier this year reported that approximately 80% of patients infected with COVID-19 showed mild symptoms or were asymptomatic, and eventually recovered without any medical intervention, whereas 15% of infected persons presented with severe illness, including shortness of breath, septic shock and multiple-organ failure, and remaining 5% of cases categorised as fatal requiring specialised care [6].

The emergence of COVID-19 in Wuhan was reported to have originated from zoonotic source but now transmitted from person to person, primarily via direct contact or through droplets spread by coughing or sneezing from an infected individual [4]. Infection of SARS-CoV-2 is facilitated by fusion of the receptor-binding domain of the viral spike proteins on its surface to the human cellular receptor identified as angiotensin-converting enzyme-2 (ACE-2) on the cell membrane, especially in the lung epithelial cells [1,7]. The study by Wang *et al*. [5] further reiterated that binding of the SARS-CoV-2 special proteins to ACE 2 is synonymous with the sequence of receptor binding of SARS-CoV. Preventive protocols have been established to halt the exposure and/or transmission of COVID-19, which include regular washing of hands with soap under running water, using face masks, practicing social distancing, and isolating suspected and confirmed cases [8]. Due to the current community transmission of COVID-19, one could easily infect him or herself; however, healthcare providers are at a higher risk of contracting the infection due to the constant exposure to patients and their specimens. The Centre for Disease Prevention and Control (CDC) confirmed in a report that in less than two months since the emergence of COVID-19, a total of 1,716 health workers had been infected with the virus, and five of them had died in China [9]. It is therefore crucial to establish the level of healthcare workers´ knowledge and attitudes during highly infectious diseases like COVID-19. Previous studies have exposed health workers´ insufficient knowledge and poor attitude toward SARS CoV [10] and MERS CoV [11]. Askarian *et al*. [12] and Sarani *et al*. [13] had also discussed the knowledge of medical staff towards infectious diseases and their willingness to work during an outbreak. Another study in 2011 revealed that only 82.3% of medical staff expressed their willingness to handle the 2009 H1N1 patients [14].

A recent study suggested the need to pay much attention to the knowledge and attitudes of medical staff at psychiatric hospitals in China during the on-going COVID-19 pandemic [15]. A similar finding was observed in a study at District 2 Hospital in Ho Chi Minh City (HCMC) which advocated the need for extensive educational interventions and campaigns for healthcare workers to be able to effectively handle COVID-19 [16]. Offinso North district is located in Ashanti region of Ghana, and has so far recorded over 798 COVID-19 cases with 6 deaths. However, no study has been conducted to assess the level of healthcare providers´ knowledge, preparedness, and willingness to handle the condition in the study area. This study therefore aimed to assess the level of healthcare workers knowledge, awareness, preparedness, and willingness to care for COVID-19 patients in Offinso North District. Findings from this study will assist authorities in planning appropriate trainings in order to furnish healthcare providers with current information on COVID-19. This will eventually enhance the willingness of health workers to care for COVID-19 patients and promote delivery of best practices to control the novel COVID-19 disease.

## Methods

**Study design and study setting:** this multi-centre, cross-sectional study was conducted in three health facilities (Nkenkaasu District Hospital, Akumadan Health Centre, and Janie Speaks A.M.E. Zion Hospital, Afrancho) in Offinso North district, between April, and May, 2020. The Offinso North District is one of twenty-seven districts in Ashanti Region of Ghana, and shares boundaries with Offinso Municipal, Tano South, Techiman South, and Afigya Kwabre East districts. The three facilities are located within peri-urban settings and Akumadan serves as both the district capital and entry point from the Ashanti Region to Bono East region. The approximately 68,543-populated district covers a total land area of about 945.9 square kilometres and lies between longitudes 10 60 W ,10 45 E and latitudes 70 20 N, 60 50 S. Farming is the most prominent work in the Offinso North district; about 552 healthcare workers of varying professions inhabit the district [17,18].

**Study participants:** the study recruited two hundred and sixty-one (261) healthcare workers from Nkenkaasu District Hospital, Akumadan Health Centre and Janie Speaks A.M.E. Zion Hospital, Afrancho between April, and May, 2020. Healthcare providers including Prescribers, medical laboratory scientists, pharmacists, nurses/midwives, public health personnel, and non-clinical staff were eligible for the study. Healthcare workers who were on leave during the study were excluded.

**Data collection and measures:** a 30-item survey questionnaire was designed, adopted from the Giao *et al*. [16] study. The questionnaires were distributed and collected through an online survey. As WhatsApp is known to be the most predominant social media platform used in the study area and Ghana at large, the questionnaire was sent to various health workers via WhatsApp platforms hosting healthcare workers practicing within the district; 261 of these filled and returned the forms to the authors between 6th and 17th April, 2020. The socio-demographic characteristics collected encompassed gender, age, education level, profession, staff category, years of work experience, and the facility of affiliation. The questionnaire had 23 other questions: 7 regarding clinical knowledge, 3 on practice of precautionary measures, 7 on preparedness, 3 on awareness of their facility´s preparedness, and 7 on misconceptions about COVID-19. A scoring system was used to analyze responses to the closed ended questions on knowledge, practice of precautionary behaviour, preparedness, awareness of preparedness, and misconception: 1 = coded as correct response and 0 = coded as incorrect response. Anyone who does not know the answer was considered to have an incorrect response. Therefore, according to this study, those scoring below the median are considered to have poor scores and above or equal to the median are considered to have good scores for the parameter under consideration.

**Statistical analysis:** analysis of data was conducted with Statistical Package for Social Sciences (SPSS) version 22.0. The statistical significance level was set at p<0.05 (two-sided). Frequencies, proportions, and graphs were used to descriptively represent knowledge, practice, preparedness, and misconceptions. Independent samples t-test, one-way analysis of variance (ANOVA), Chi-square test, or correlation test were used appropriately to compare knowledge, practice and preparedness scores of the different participants according to demographic characteristics. Binary logistic regression analyses were used to identify factors associated with knowledge, practice and preparedness. Factors were selected with a backward stepwise method. Odds ratios (ORs) and 95% confidence intervals (CIs) were used to quantify the associations between variables and knowledge, practice and preparedness.

**Ethical consideration:** we followed strictly “Declaration of Helsinki-Ethical Principles for Medical Research” throughout the study. This study was approved by the Offinso North District Health Directorate. This directorate oversees all health-related activities within the district. Approvals from managements of the three facilities were also sought for before commencement of this study. The eligible healthcare workers were duly informed of the objectives of the study, and they consented to it before participation. Participants were assured of the confidentiality of the data provided as their responses were anonymous.

## Results

**Demographic characteristics of study participants:** Table 1 depicts the main sociodemographic characteristics of the study participants. The questionnaire was completed by a total of 261 participants. The average age was 32.0±6.30 years, with a minimum of 20 years and a maximum of 59 years. Males were 132 (50.6%) and 129 (49.4%) were females. Most of the participants held Diploma/HND (52.7%, n=137), were clinical staff (75.5%, n=197) and Nurses/Midwives (56.7%, n=148). Nearly 48% of the health workers had <5years working experience, and majority (54.8%, n= 143) of them were from Nkenkaasu District Hospital (Table 1). The knowledge scores of the participants significantly varied across age groups (p=0.001), education (p<0.001), staff category (p=0.001) and profession (p<0.001). The score for the practice of precautionary measures significantly differed across educational levels (p=0.001) and profession (p=0.038). Health workers´ preparedness significantly varied across gender (p=0.025), age (p=0.001), education level (p=0.001), profession (p=0.013) and job category (p=0.016).

**Table 1:** Demographic characteristics of participants and knowledge, practice, and preparedness scores of COVID-19 by demographic variables

Characteristics			Knowledge score			Practice score			Preparedness score		
		N (%)	Mean±SD	t/F	P	Mean±SD	t/F	P	Mean±SD	t/F	P
**Gender**	Male	132(50.6)	7.21±2.15	1.138	0.256	4.50±1.17	0.259	0.813	4.01±1.43	2.259	0.025
	Female	129(49.4)	6.95±1.60			4.47±1.21			3.63±1.28		
**Age (years)**	20-29	102(39.2)	6.99±1.20	5.841	0.001	4.36±1.16	0.937	0.423	3.82±1.28	5.743	0.001
	30-39	137(52.7)	6.89±2.23			4.53±1.22			3.64±1.39		
	40-49	12(4.6)	8.25±1.71			4.58±1.00			4.83±0.94		
	50-59	10(3.8)	9.25±1.38			5.00±1.31			5.13±1.55		
**Education level**	PhD/Masters	8(3.1)	8.88±2.85	5.879	<0.001	4.00±1.31	4.664	0.001	4.63±1.85	4.867	0.001
	Bachelor	61(23.4)	7.08±1.94			4.23±1.35			4.07±1.31		
	Diploma/HND	121(46.4)	7.21±1.61			4.56±1.15			3.92±1.44		
	Certificate	64(24.5)	6.91±1.96			4.78±0.95			3.47±1.04		
	SSSCE/BECE	7(2.7)	4.43±1.98			3.14±0.90			2.29±1.11		
**Staff category**	Clinical	197(75.5)	7.37±1.50	3.375	0.001	4.55±1.14	1.573	0.119	3.94±1.33	2.494	0.013
	Non-Clinical	64(24.5)	6.20±2.62			4.27±1.31			3.45±1.41		
**Profession**	Nurse/Midwife	148(56.7)	7.33±1.40	8.818	<0.001	4.64±1.06	2.395	0.038	4.01±1.34	2.860	0.016
	Prescribers	7(2.7)	8.57±1.40			5.00±0.00			4.43±0.53		
	Pharmacist	10(3.8)	8.00±1.49			4.80±0.79			4.10±1.73		
	Med. Lab. Sci.	30(11.5)	7.20±1.77			4.03±1.52			3.73±1.31		
	Public Health	19(7.3)	7.53±1.74			4.16±1.12			3.37±1.26		
	Others	47(18.0)	5.62±2.70			4.28±1.38			3.37±1.26		
**Work experience**	<5 years	124(47.5)	7.06±1.37	2.239	0.109	4.48±1.19	0.230	0.795	3.90±1.30	2.750	0.066
	5-10 years	108(41.4)	6.93±2.22			4.45±1.23			3.62±1.30		
	>10 years	29(11.1)	7.76±2.42			4.62±1.01			4.24±1.77		
**Facility**	Nkenkaasu	143(54.8)	6.87±1.74	3.030	0.050	4.48±1.10	0.230	0.795	3.87±1.41	0.734	0.481
	Akumadan	48(18.4)	7.65±2.44			4.48±1.27			3.60±1.27		
	A.M.E Zion	70(26.8)	7.11±1.72			4.49±1.30			3.86±1.34		

Med.Lab.Sci.=Medical Laboratory Scientist; SD= Standard deviation

**Demographic characteristics of study participants:** Table 1 depicts the main sociodemographic characteristics of the study participants. The questionnaire was completed by a total of 261 participants. The average age was 32.0±6.30 years, with a minimum of 20 years and a maximum of 59 years. Males were 132 (50.6%) and 129 (49.4%) were females. Most of the participants held Diploma/HND (52.7%, n=137), were clinical staff (75.5%, n=197) and Nurses/Midwives (56.7%, n=148). Nearly 48% of the health workers had <5years working experience, and majority (54.8%, n= 143) of them were from Nkenkaasu District Hospital (Table 1). The knowledge scores of the participants significantly varied across age groups (p=0.001), education (p<0.001), staff category (p=0.001) and profession (p<0.001). The score for the practice of precautionary measures significantly differed across educational levels (p=0.001) and profession (p=0.038). Health workers´ preparedness significantly varied across gender (p=0.025), age (p=0.001), education level (p=0.001), profession (p=0.013) and job category (p=0.016).

**Figure 1:**
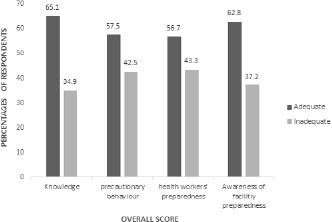
Knowledge, practice and preparedness of the participants

**The overall knowledge, practice, preparedness, and misconceptions of the participants:** generally, 65.1% of the respondents had adequate knowledge about the COVID-19 outbreak; 57.5% practiced precautionary behaviour adequately. Almost 57% of the participants were adequately prepared to handle cases, and 63% were aware of their facility´s preparedness for the COVID-19 (Figure 1). Finally, the overall misconception about the COVID-19 disease among the health workers was high (62.5%)

**Figure 2:**
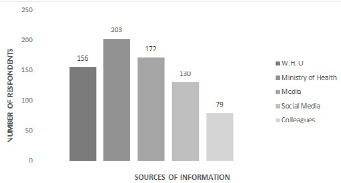
Sources of information about COVID-19 among the study population

**Sources of information about the COVID-19:** the majority 203/261 (77.78%) received information through the Ministry of Health platform, 172/261 (65.9%) reported to have received their information from the media house publications (television, radio and newspaper). Others stated World Health Organization (156/261, 59.77%), social media (130/261, 49.81%) and their colleagues (79/261, 30.27%) as their sources of information (Figure 2).

**Factors significantly associated with COVID-19 inadequate knowledge and preparedness:** the binary logistic regression analysis revealed that education level (PhD/Masters vs. SSCE/BECE [OR: 18.00, p=0.033]), staff category (clinical vs. non-clinical [OR: 0.359, p=0.001]) and profession (nurse/midwife vs. others [OR: 3.599, p<0.001]) were significantly associated with inadequate knowledge of health workers on COVID-19. Similarly, education levels of PhD or masters (vs. SSCE/BECE, OR: 18.00, p=0.033), profession of clinical staff (vs. non-clinical, OR: 2.593, p=0.001) and nurses/midwives (vs. non-clinical staff, OR: 3.599, p≤0.001), were significantly associated with lower knowledge scores (Table 2).

**Table 2:** Results of binary logistic regression analysis on factors significantly associated with COVID-19 inadequate knowledge and preparedness

Variable	OR (95%CI)	p-value
**Knowledge**		
Education (SSSCE/BECE vs. PhD/Masters)	18.00 (1.267-255.744)	0.033
Staff category (Clinical vs. Non-clinical)	0.359 (0.201-0.641)	0.001
Profession (Non-clinical vs. Nurses/Midwives)	3.599 (1.819-7.118)	<0.001
**Preparedness**		
Education (SSSCE/BECE vs. PhD/Masters)	18.00 (1.267-255.744)	0.033
Staff category (Clinical vs. Non-clinical)	2.593 (1.453-4.627)	0.001
Profession (Non-clinical vs. Nurses/Midwives)	3.276 (1.644-6.527)	0.001

**Associations between the variables of interest:** the results from the bivariate correlation analysis showed a significant positive linear relationship between the participants´ knowledge about the Novel Corona virus disease and their preparedness (r= 0.285, p= <0.001), awareness of the facilities preparedness (r=0.162, p=0.009), and misconception (r=0.150, p=0.016). Also, the practice of precautionary measures and preparedness for possible cases were positively related to the awareness of the facility´s preparedness towards the disease (r= 0.181, p= 0.003) and (r= 0.208, p= 0.001) respectively (Table 3).

**Table 3:** Bivariate correlations of knowledge about the COVID-19, practice, preparedness, awareness of facility preparedness and misconceptions

Variable	N	r	p
Knowledge vs. preparedness	261	0.285	<0.001
Knowledge vs. awareness	261	0.162	0.009
Knowledge vs. misconception	261	0.150	0.016
Practice vs. awareness	261	0.181	0.003
Preparedness vs. awareness	261	0.208	0.001

## Discussion

The current coronavirus disease has had a devastating effect globally, ever since it was declared a public health emergency by WHO. Ghana recorded its first case on March 12, 2020, and since then, the number of new cases has consistently been in ascendency. This poses a serious occupational health risk to health workers due to their frequent exposures to infected individuals and their specimens [19]. Adequate knowledge, good practice of precautionary measures, adequate preparedness and as well as absence of misconception are crucial in efficient management of infected patients with minimum risk predisposition [20]. Therefore, assessment of workers´ knowledge, awareness, preparedness and misconceptions towards COVID-19 during the rapid rise period of the outbreak is vital in the fight against the pandemic. To the best of our knowledge, this study is the first to analyse the level of knowledge, practice, preparedness, and misconceptions on COVID-19 among healthcare workers in Ghana. The present study showed that, 65.1% of healthcare providers in the three facilities had adequate knowledge about the pandemic, and 27.6% of them had received appropriate in-service training. Also 57.5% of the respondents were adequately prepared, and were willing to care for COVID-19 patients should the district confirm a case, and 63.0% of them were duly aware of their facility´s preparedness to manage cases. Again, only 57.5% of the health workers adhered strictly to the practice of precautionary measures, and more than half (62.5%) of the participants had varying forms of misconceptions about the aetiology of COVID-19.

The level of knowledge exhibited by the healthcare providers in this study is incomparable to previous studies during disease outbreaks. A recent study in psychiatric hospitals in China demonstrated that 89.51% of the medical staff had extensive knowledge of COVID-19 [15], and Giao *et al*. [16] also observed similarly higher level of knowledge on the current pandemic. The difference in knowledge gap may be due to the fact that only few (27.6%) of our respondents had received relevant training on COVID-19, whiles more than half of participants in the Shi *et al*. [15] study had obtained formal training on the outbreak. Earlier studies have stressed the relevance of in-service training by health facilities and other agencies in the prevention of contagious diseases [21-24]. It is very essential to strengthen healthcare workers to enable them attain the knowledge and skills required to understand and practice evidence-based medicine [25]. Again, the acquisition of knowledge on infectious diseases like COVID-19 is clinically significant, especially in present state of affairs when vaccines or potent medications are unavailable; this will enable health workers receive up-to-date information and take all necessary precautions during supportive treatment and prevention of the infection.

Stergachis *et al*. [26] reiterated in their study that the willingness of workers to work is enhanced by the implementation of relevant educational programmes and protective procedures. Another study showed that progressive training was an independent factor linked with a higher likelihood of health workers´ (77.17%) willingness to handle psychiatric patients with COVID-19 [15]. It is therefore not surprising to witness that only a little over a half of the health workers in this study were willing to care for patients who may be suffering from COVID-19. Again, in 2009, over 82% of health workers in China were willing to take care of people infected with the relatively less infectious and less fatal H1N1 [14]. Due to the fact that the novel COVID-19 is still under study and much is not known about the properties of the virus [15], most healthcare providers are more particular about infecting themselves and their relatives. In addition, inadequate personal protective equipment (PPEs) is a common occurrence in the hospitals of resource-limited countries like Ghana, and this demotivates workers to compromise and put in their best at work. The above details may describe why over 40% of the interviewed health personnel in Offinso North district were unwilling to attend to patients who may be suffering from COVID 19. We advocate that authorities facilitate progressive training for staff on COVID-19 in order to enhance the workers´ willingness to care for COVID-19 patients.

The present study showed that 57.5 % of the participants practiced adequate precautionary behaviours like wearing of mask (77.8%) and frequent washing of hands with appropriate soap and water or rubbing alcohol-based sanitizer (99.2%), however, an appreciable proportion of the health workers (45.2%) used gloves/goggles only when the case is suspected to be COVID-19. The level of practice of precautionary measures identified in this study could be related to the level of knowledge these professionals had on the COVID-19. Again, most of the healthcare workers had idea regarding the high infectivity of SARS-CoV-2, which can be easily transmitted between people via the invisible respiratory droplets, and this might have contributed to their adequate practice of the preventive behaviours. Giao *et al*. [16] and Bhagavathula *et al*. [19] also reported that majority of the health workers had positive attitudes towards COVID-19, a measure based on practice of precautionary measures. There was significant difference between education level, profession, and practice of precautionary measures (p<0.05) while there was no significant difference between gender, age, staff category, and experience (p>0.05). The [16] study also found that attitude regarding COVID-19 had no association with age, gender, and experience but found statistically significant association with occupation/job.

Our findings revealed adequate (57%) preparedness of health workers towards possible cases of the diseases, and 63% of them were aware of their facility´s preparedness for COVID-19 management. The relatively good knowledge of the healthcare workers and their sufficient level of the practice of precautionary behaviours could have accounted for their sense of preparedness. Correlational analysis also augments the above findings from the present study, as a significant positive linear relationship was seen between the participants´ knowledge and their preparedness (r= 0.285, p= <0.001) and awareness of the facilities´ preparedness and their preparedness (r= 0.162, p=0.009). This correlation could also be explained by the Reasoned action theory which stresses that a person´s intention to a specific behaviour is a function of their attitude towards that behaviour [27]. Similarly, the practice of precautionary measures (r= 0.181, p= 0.003) and health workers´ awareness of the facility´s preparedness to fight the disease (r= 0.208, p= 0.001) positively correlated with their preparedness towards possible cases of COVID-19 in the district. These findings clearly indicate the importance of providing adequate personal protective equipment (PPEs) and equipping hospital facilities which may also result in improvements in their preparedness. Preparedness scores significantly differed across gender, age-groups, education, staff category, and profession (P<0.05).

Health workers who had attained SSCE/BECE education had higher odds of having inadequate knowledge compared with the reference category of those with PhD/Master degree (OR: 18.00, p=0.033). Similarly, clinical staff were 0.359 times less likely to have inadequate knowledge about COVID-19 compared with the non-clinical staff (OR: 0.359, p=0.001). In addition, non-clinical health workers in the facilities had higher odds of having inadequate knowledge compared with nurses/midwives (OR: 3.599, p<0.001). The findings also indicated that health workers who had attained SSCE/BECE education had higher odds of having inadequate preparedness compared to those with higher education (OR: 18.00, p=0.033); clinical staff were 2.59 times more likely to be inadequately prepared to care for COVID-19 patients as compared with non-clinical staff (OR: 2.593, p=0.001). Furthermore, the non-clinical health workers were less likely to attend to suspected or confirmed COVID-19 of patients compared with nurses/midwives (OR: 3.599, p≤0.001). The revelation of higher level of COVID-19 knowledge and preparedness in people of higher educational background was eminent in an online survey among Chinese residents during the current pandemic [28]. The difference could be related to the fact that graduate programmes are often partly or completely research-based, and prepare individuals to develop the habits of looking for information about any emerging condition. Reception of better knowledge of the COVID-19 directly influences their enhanced preparedness to receive and care for the affected ones. We identified that the respondents realised the pandemic COVID-19 was a global threat and obtained information on it via diverse sources such as the Ministry of Health (77.78%), WHO (59.77%), social media (49.81%), colleagues (30.27%) and media houses (46.90%). The higher percentage of COVID-19 updates from the Ministry of Health was influenced by the Government of Ghana´s decision to address the nation regularly on the pandemic-related issues. This is contrary to the study by Giao *et al*. [16] where social media was the major source of information on evolving infectious diseases like COVID-19 in Vietnam. Another study also found health workers´ main source of information during the MERS-CoV outbreak to be through seminars and workshops [29].

There has also been a quantum of misinformation regarding COVID-19; which scientists and WHO suggest might lead to xenophobic attacks in the world [30,31]. Also, there is over-abundance of mischievous and unconfirmed reports waved on the internet that spread quickly, and could misguide health workers. The overall misconception about COVID-19 among the health workers in our study was higher (62.5%) than expected despite the fact that a good proportion of the health workers were clinical staff that had completed higher education and expected to be enlightened. It is required of health workers to carefully evaluate their sources of information about the pandemic and utilize accurate content to seek information in the practice of present-day evidence-based medicine [19]. This would probably alleviate the stigmatization related to the aetiology, prevention, and care of COVID-19 patients. This study was not without limitations. The administration of the questionnaire through WhatsApp messenger, the mostly used social media platform in Ghana, could not exclude the likely influence of selection bias. Again, the self-reported questionnaire we employed in the study might not signify real practice.

## Conclusion

Even though health workers´ knowledge and preparedness towards COVID-19 were encouraging, this study still elucidates the knowledge gap of the professionals on the novel pandemic in Offinso North district. The high sense of misconception on COVID-19 than expected among the healthcare providers could be detrimental in the care of patients, and may promote stigmatization. We advocate for progressive training of staff on COVID-19, to advance the appreciation of the risks and precautionary measures and clear the misconceptions among essential health workers in the battle against the current deadly pandemic. This, we believe, would boost their confidence and increase their willingness to deliver efficiently.

### What is known about this topic

- Coronavirus disease 2019 (COVID-19) is recognized as global pandemic;
- Covid-19 outbreak was unique in terms of high pathogenicity and mortality compared to the previous epidemics by coronaviruses;
- No data exist on knowledge, practice and preparedness among health workers to respond the COVID-19 in Ghana and West Africa.

### What this study adds

- Knowledge, practice and preparedness among health workers and their main source of information;
- There was adequate Knowledge, practice and preparedness to prevent and control COVID-19;
- Findings indicate the need for ministry of health to timely and effectively disseminate Covid-19 prevention and control measures to Health workers in Ghana.

## Competing interests

The authors declare no competing interests.
